# An experimental study of pathologist's navigation patterns in virtual microscopy

**DOI:** 10.1186/1746-1596-5-71

**Published:** 2010-11-18

**Authors:** Lucia Roa-Peña, Francisco Gómez, Eduardo Romero

**Affiliations:** 1 Department of Pathology, School of Medicine, National University of Colombia, Bogotá, Colombia; 2 Bioingenium Research Group, School of Medicine, National University of Colombia, Bogotá, Colombia

## Abstract

In virtual microscopy, a sequential process of captures of microscopical fields, allows to construct a virtual slide which is visualized using a specialized software, called the virtual microscopy viewer. This tool allows useful exploration of images, composed of thousands of microscopical fields of view at different levels of magnification, emulating an actual microscopical examination. The aim of this study was to establish the main pathologist's navigation patterns when exploring virtual microscopy slides, using a graphical user interface, adapted to the pathologist's workflow. Four pathologists with a similar level of experience, graduated from the same pathology program, navigated six virtual slides. Different issues were evaluated, namely, the percentage of common visited image regions, the time spent at each and its coincidence level, that is to say, the region of interest location. In addition, navigation patterns were also assessed, i.e., mouse movement velocities and linearity of the diagnostic paths. Results suggest that regions of interest are determined by a complex combination of the visited area, the time spent at each visit and the coincidence level among pathologists. Additionally, linear trajectories and particular velocity patterns were found for the registered diagnostic paths.

## Background

A very recent field, known as virtual microscopy, has made possible digital exploration of histological slides, archiving of these slides for later analysis and easy access to this information. A slide digitization is called a virtual slide (VS) and is constructed from sequential captures of microscopical fields [1]. These virtual slides are high resolution images whose visualization requires a specialized software, called the virtual microscopy viewer, a specific tool devised for running over images composed of thousands of microscopical fields of view.

Complex mechanisms are involved in the genesis of the navigation patterns, guided by the training time and triggered by the contents of the slide [2]. Coarsely, diagnosis in pathology can be considered as a process composed of four sequential steps: look, see, recognize and understand [3]. A definitive diagnosis is achieved by following a standard methodology with two coarse phases: first examination is carried out at the lower magnification (panoramic) in order to locate relevant information in terms of a spatial organization of the histological sample (scanning), while the second and further examination is conducted for analysis of the slide contents which implies changing the magnification (zoom) [3-5]. This analysis is performed through navigation of the zoomed areas [5], on which gentle movements are generally required. This learned strategy has been observed in multiple studies in which it has been possible to determine the existence of these two phases. Tsuchihashi et al. studied one pathologist exploring twenty different slides in telepathology. This investigation identified two patterns: exploration at low magnification and analysis at higher magnifications [4]. Crowley et al. recorded on videotape diagnoses performed on four histological slides by fifteen pathologists, distributed into three different categories: novices, intermediates and experts [5]. Results showed that intermediates and experts exhibited very similar patterns to the two described before, i.e., a general search strategy and selection of areas to revisit them at higher power. Tiersma et al. investigated visual exploration patterns in pathology using an eye tracker mechanism.

Results showed two patterns: scanning (saccadic eye movements) and selection (eye fixation over specific points for further exploration) [6]. Finally, Krupinski et al., using an eye tracker system in a group of pathologists, demonstrated that visual exploration is characterized by a rapid determination of ROIs, which likely contain diagnostic information [2]. It is worthy to strengthen out that the experimental setup of that study allowed only scanning patterns, i.e., magnification changes were not available.

Overall, the experience with images in virtual microscopy should be very similar to an actual optical examination. Therefore, design of friendly and useful graphical user interfaces (GUIs) is a fundamental issue. Indeed, navigation patterns arise from two intermixed processes: the motor control associated to some movement automations and a refined search information process, which reflects the level of expertise. Yet patterns may be different, the more expert is the group of pathologist the more similar are the locations they visit when exploring a histological slide [2]. It is important to keep in mind that the virtual microscopy tool does not imitate exactly a conventional light microscope, but rather it has the purpose of allowing a pathologist to navigate at any resolution, while the VS is always available at the lowest resolution, that is to say, the expert always conserves a thorough panorama of the VS.

This study aims to determine the main factors involved in the genesis of the navigation patterns from a particular diagnostic path. These patterns are the result of the interaction between the image contents and the expert experience. Other studies have focused before on studying either the attentional mechanisms that guide trajectories in this type of images, or the general mechanisms at the very base of the interaction of an expert with an image. On the contrary, our study integrates these two visions and introduces new elements as the magnification changes. Finally, as far as we know, this is the first study which actually evaluates how a pathologist *moves the stage*, in other words, we dedicated our endeavors to figure out the influence of both the image contents and the pathologist methodology in an actual interaction context.

## Methods

A total of four randomly selected histological specimens were digitized and six VS were assembled, using an acquisition system composed of a Carl Zeiss Axiostar Plus microscope, a Sony high resolution digital video camera Handycam DCR-HC85 (640 × 480 pixels) and two Carl Zeiss adapters: 426126 and 456006 (Carl Zeiss, Light Microscopy, Gottingen, Germany). Hematoxylin-eosin tissue samples from endomyometrium, gallbladder, prostate and a uterus leiomyoma were used for this study.

Histopathological slides were selected from a set of routine cases from the Pathology Department at the National University of Colombia, a laboratory of medium complexity. These samples were selected by an expert pathologist. The endomyometrium and the leyomioma were obtained from a total hysterectomy of a patient with abnormal uterine hemorrhage due to multiple leyomiomas; the endomyometrium had only focal epithelial componet and the leyomyoma was basically composed of smooth muscular tissue with quite homogeneous distribution. The gallbladder specimen was obtained from a colecistectomy and the prostate sample was obtained by transrectal biopsy, case in which the tissue is characterized by the presence of glands supported by stroma. Finally, the uterus leiomyoma, obtained from a myomectomy, is basically composed of a muscular tissue whose distribution is quite homogeneous. They were digitized and six different images were assembled. Sizes in pixels were 53280 × 39360, 42480 × 15840, 33840 × 21600, 53280 × 39360, 42480 × 15840 and 49680 × 28320 which stand for an effective area of 11.97 × 8.84, 9.54 × 3.56, 11.16 × 6.36, 11.97 × 8.84, 9.54 × 3.56, 11.16 × 6.36 *mm*^2 ^(pixel size of 1.98 *μm*^2^), respectively. Mega-images were stitched using automatic registration with cross correlation as the similarity measure and were stored in JPEG2000 format for latter access and navigation [7].

### GUI design

Pathologist navigation patterns were recorded using a virtual microscope prototype whose GUI was adapted to the pathologist requirements [7]. This design exploits the importance of low magnifications for exploration and analysis at high resolutions for diagnosis. The GUI is composed of a thumbnail and an auxiliary window. The former displays the lowest resolution thumbnail image, in which a rectangular re-sizable window allows a required selection. The thumbnail window is set to a desired size at the beginning, while the auxiliary window is constantly varying, according to the magnification level of the selected ROI in the thumbnail window. Displacements of a particular ROI were only allowed in the thumbnail window through drag and drop operations. Finally, for each requested ROI, its position, size, resolution and time were recorded for later analysis.

**Figure 1:**
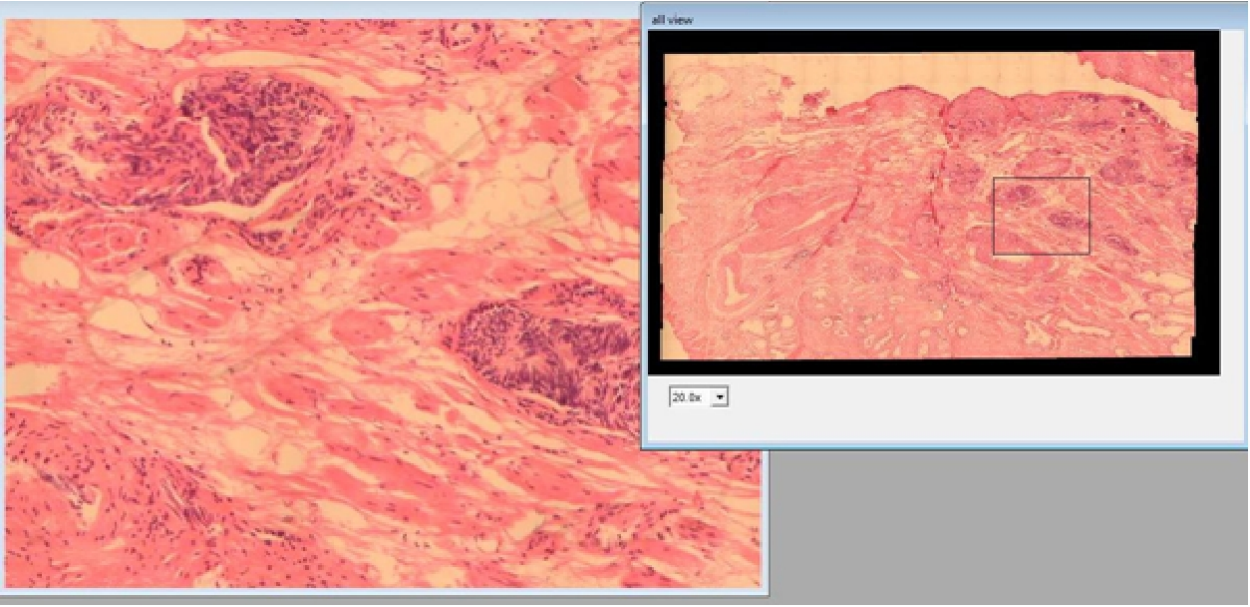
**GUI of the virtual microscopy prototype**. GUI of the virtual microscopy prototype: the figure shows the whole Virtual slide at the right panel (small magnification) while a large resolution of the black square in the VS enlargement is displayed in the auxiliary window at the left panel. Panning was only allowed in the VS window.

Figure 1 shows the virtual microscope GUI. Navigation in the developed prototype is carried out through a conventional mouse and consists of two processes: first, a window is picked at the thumbnail image (the VS), followed by a displacement of this window to an interest point, proportional to the mouse movement. This prototype was aimed at achieving integration of this kind of tools with a routine pathologist's work, a design that should allow simultaneous displays at different magnifications.

### Experimental setup

The aim of the present study was to compare navigation patterns when expert pathologists are exploring virtual slides. Four expert pathologists participated in this study, all of them had similar years of experience (about five years), and were graduated from the same pathology school program. Each pathologist was previously trained on virtual microscopy using two test virtual slides. Average, the training time was 20 minutes so that at the end of this time they were free to navigate the slides at will, until they could reach a probable diagnosis and organ identification. Six virtual slides were chosen, as parts of full histological slides, with a relative size which varied between 10% to a 30% of the whole histological sample. An experienced pathologist, with at least five years of experience, selected the digitized area. For the sake of the experiment, images shown to the pathologist belonged to areas in which it was difficult to determine both the organ and the pathological entity so they were forced to spend more time, exploiting the navigation tool [8].

The six VS were randomly displayed for each of the pathologists so that the examination order was always different. Each pathologist was asked to run over the virtual slide, up to a diagnosis was set, using the same screen monitor they use in their routine computer work (CCFL (220 nits) WXGA (1280 × 800 of 13, 3")). During examinations, every pathologist action was recorded for later analysis, namely, we recorded the ROI location, the ROI size related to the thumbnail image, the time any action (drop or drag) was carried out and the magnification level.

### Evaluation issues

We claim that from a navigation point of view, an image can be thought of as an ordered partition of spatial locations with different levels of relevance associated to each. Therefore, an image is composed of regions with different levels of interest. Pathologists shall visit a minimum number of regions, thereby gaining a maximal amount of information in a minimal time. Evaluation was then addressed to verify these two main issues: images are composed of pieces of information with different levels of relevance (Regions of Interests) and pathologists will use a minimum time exploring them (Navigation Patterns). The first item was assessed as follows:

1. The percentage of visited area was calculated for the group of pathologists and the whole set of VS.

2. The percentage of coincidence among the different visited areas, i.e., at least two pathologists examined the same image region within the navigation.

3. For each pathologist, the time spent per region was also computed.

4. Coincidence among ROIs, defined as the common visited areas and longer visits was calculated.

The second item was assessed as follows:

1. Mouse movement velocities were registered and analyzed.

2. Linearity of trajectories: Euclidian distance was computed and compared against the actual trajectory distance, i.e., a ratio between the two distances was calculated.

## Results

### Regions of Interests

As previously discussed, we suppose that ROIs are defined by a combination of the three criteria presented hereafter:

#### Percentage of visited area

The percentage of visited area by at least one pathologist was computed for each of the virtual slides (Table 1). These values varied between 44% and 91% with an average of 66%, indicating that the amount of explored tissue is highly dependent on the image contents, that is to say, some virtual slides were little-explored because relevant information was easily available.

**Table 1:** Percentage of visited area for the group of pathologists in the whole set of VSs.

Image	Percentage of visited area per image
image 1	91%

image 2	48%

image 3	67%

image 4	86%

image 5	44%

image 6	60%

Pathologists were forced to further explore the image, attempting a maximum level of information, but in general this was hardly established since information was not enough as to consolidate a diagnosis. Results support this statement since the percentage of explored image was larger than a 50%.

#### Percentage of Coincidence

The percentage of coincidence of the visited areas among pathologists of each VS was computed (Table 2). These values varied between 41% and 97%, with an average of 70.5%, indicating that the explored areas were quite similar, though the VS content is entirely different. The coincidence level turns out to be dependent on the kind of information present in the virtual slide and located in specific regions. For example, table 1 shows that pathologists visited a 48% of the 2th VS, while its level of coincidence was 97%. In this virtual slide it is observed that there is no tissue in about a 30% of the entire VS. Interestingly, the histological sample corresponds to an endo-myometrium, in which the glands are the fundamental part of any diagnosis and in the virtual slide they are located in specific areas. The diagnostic path, in this case, searched these structures all over the virtual slide and overall, the four pathologists run over the same parts of the VS.

**Table 2:** Influence of the image contents in the navigation pattern observed from our experiments was assessed by measuring the coincidence level in the visited regions, namely, the area percentage which was visited by more of one pathologist.

Image	Percentage of coincidence in visited areas
image 1	63%

image 2	97%

image 3	95%

image 4	69%

image 5	41%

image 6	58%

Coincidence average was 70.5%, which demonstrates that there are relevant information areas.

As expected, samples where information is located show higher levels of coincidence. The first virtual slide corresponds to prostatic tissue, in which there is a large number of glands, so pathologists dedicated most of the navigation exploring them: the coincidence level is high (larger than 50%). The second virtual slide corresponds to an endomyometrial sample, its epithelial component is quite located and constitutes a very small area of the sample so the coincidence level is also high. The third virtual slide corresponds to a fragment of a leiomyoma with a predominant stromal component. The areas visited by the pathologists corresponded to structures with a luminal space, seen at the low magnification. Interestingly, as it was not clear if they corresponded to glands or vessels, the resultant coincidence level was again high. The fourth virtual slide was clearly a leiomyoma and yet the visited area was large (86%), the coincidence level was only 69%. The fifth virtual slide was a gallbladder, the epithelial component is minimum but scattered and then the area to explore large, the coincidence level was smaller than the other images. Finally, the sixth virtual slide corresponded to a prostate, in which the epithelial component was sparse and hence also the coincidence level and the area to explore.

#### Spent Time

A potential ROI could arise either when every pathologist stops at particular image location and therefore information therein is relevant, or when the pathologist spans a longer period in a precise area so that even if the interest in the region is not shared among the group of experts, there exists a potential source of knowledge. In consequence, we also evaluated the time every pathologist required for examining regions as the total time of visit per pixel in the thumbnail window, which was estimated by accumulating the set of visit times and computing their average. A ROI (in the time sense) was then defined as the region composed of those pixels for which this quantity was larger than the mean.

**Figure 2:**
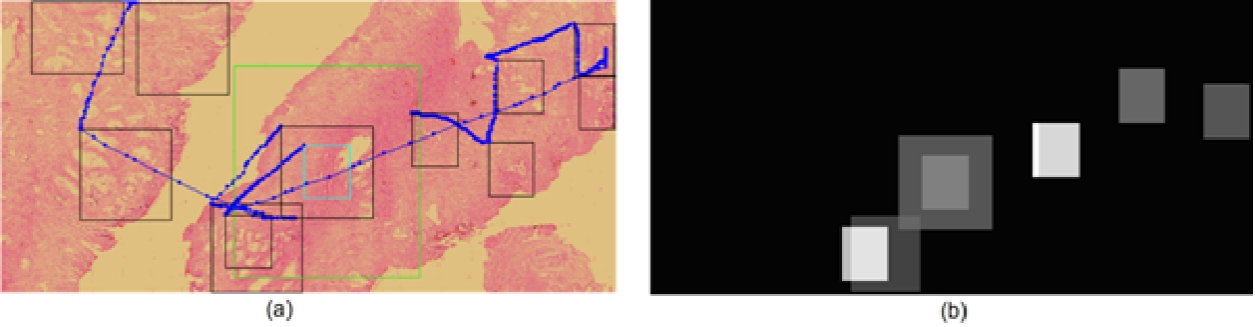
**Diagnostic path**. Left panel a whole diagnostic path composed of multiples jumps among different image locations. This diagnostic path shows not only scanning patterns at the beginning, but also magnification changes, highlighted in the figure as the green and blue squares. Once the magnification changes are established, navigation keeps under scanning patterns and the observation window is smaller since resolution is higher. At the right panel it is displayed the image locations with higher time rates. The white squares correspond to longer times while grey ones stand for smaller. Note that longer times are spent in the part of the diagnostic path which was conducted at the higher magnifications.

The analysis in this section was pointed out to determine whether or not there exists any pattern regarding the time used for analysis. Therefore, a diagnostic path could be set not only in terms of the image contents, but also according to the time a pathologist needs to explore the VS. Figure 2 illustrates a thorough diagnostic path in a VS, a scanning pattern with two different magnifications. The magnification changes are highlighted in the image as the green and blue squares. There are two scanning patterns, each at a different resolution. Interestingly, the scanning pattern at higher magnification required also higher time rates, indicating that once the search has been established, these experts devoted their efforts to analysis and diagnosis on regions in the image that contain relevant information. This analysis was extended to the entire set of pathologists.

**Figure 3:**
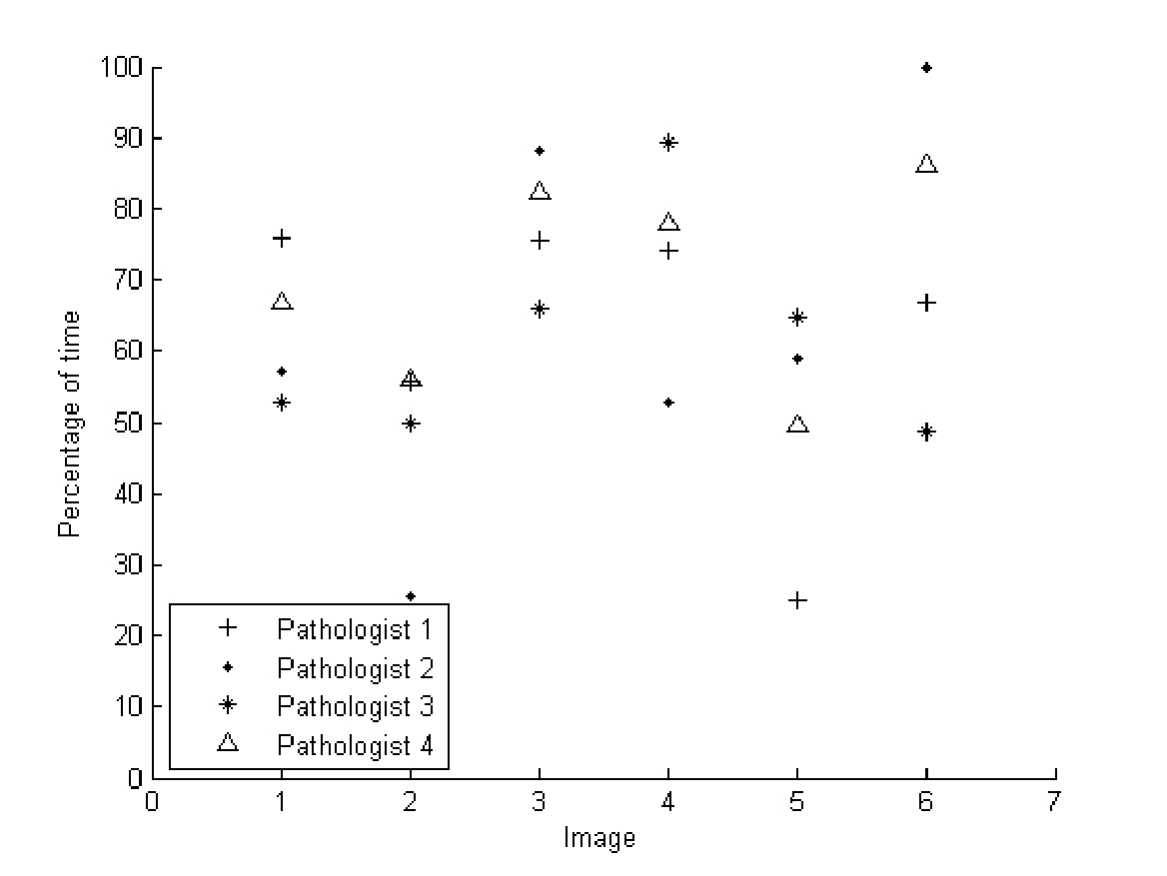
**Average time percentage spent in different ROIs**. It is displayed the average time percentage spent among the different ROIs for each of the pathologists and through the set of six images. Total times were normalized by the maximum visited time for comparing navigations with different durations. The x axis corresponds to the set of available images while the y axis stands for the average of percentage of time spent in the previously determined ROIs. Note that in general pathologists spend more than a 50% of the navigation time exploring these regions.

Figure 3 shows the percentage time every pathologist spent over the ROIs previously established at any of the six images. Overall, pathologists spent at least a 50% of the average of the navigation time on these ROIs, most of them detected at the larger magnification. The plot shows that VS two and five were less explored regarding these regions. Overall, in despite of the different contents in these images, these results indicate that pathologists spend most of the navigation in regions where information is more relevant and the scanning process turns out to be dedicated to search such information.

#### Coincidence in ROIs

One important question we addressed consisted in determining, whether or not the ROIs defined by spatial preferences or time, would coincide. A standard measure of the degree of intersection between regions was used: the Jaccard coefficient, a measurement of the similarity between sample sets, defined as the intersection divided by the union of the sample sets. This coefficient has a maximum value of 1 when there is total agreement and zero when there is none.

**Figure 4:**
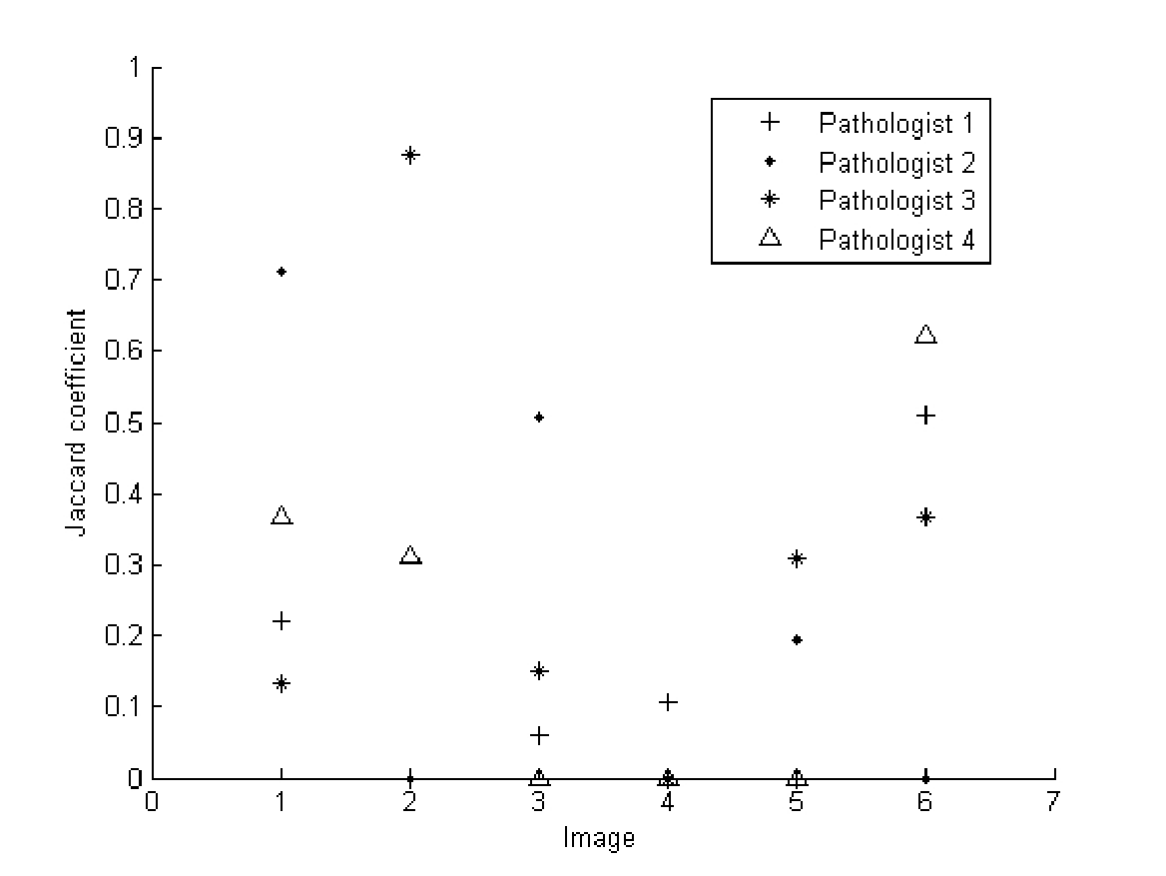
**Coincidence level between two different ROI types**. Jaccard coefficient is shown in the y-axis against the particular VS in the x-axis. This coefficient measures the level of coincidence between the two sorts of ROIs, namely, the ones determined by the number of visits and those established when a pathologist spent a significant time exploring them. In general, there is not a systematic trend and pathologists use different navigation patterns regarding times and preferred locations. These results indicate that ROIs may be defined, depending on the application, by two different sets of features.

The coefficient was thus calculated for the six VS, showing different degrees of overlapping, from a 0.9 of the third pathologist for the second image to a zero overlap coefficient for at least one of the pathologists along the whole sets of images. Overall, Figure 4 shows coincidence levels below 0.5 in most images, indicating that effectively there exist different ROIs per pathologist, defined by the spent time or by the number of visits, i.e., the level of coincidence is low. Therefore, there is no specific pattern, for instance the fourth pathologist (triangle) has no coincidence level in images three, four and five, while in images one, two and six, the Jaccard coefficient is 0.3, 0.4 and 0.6, respectively. Similar results were also observed for the pathologists, indicating that each pathologist has different preferences when searching further information, either by time or preferred location.

### Navigation patterns

#### Mouse Movements

**Figure 5:**
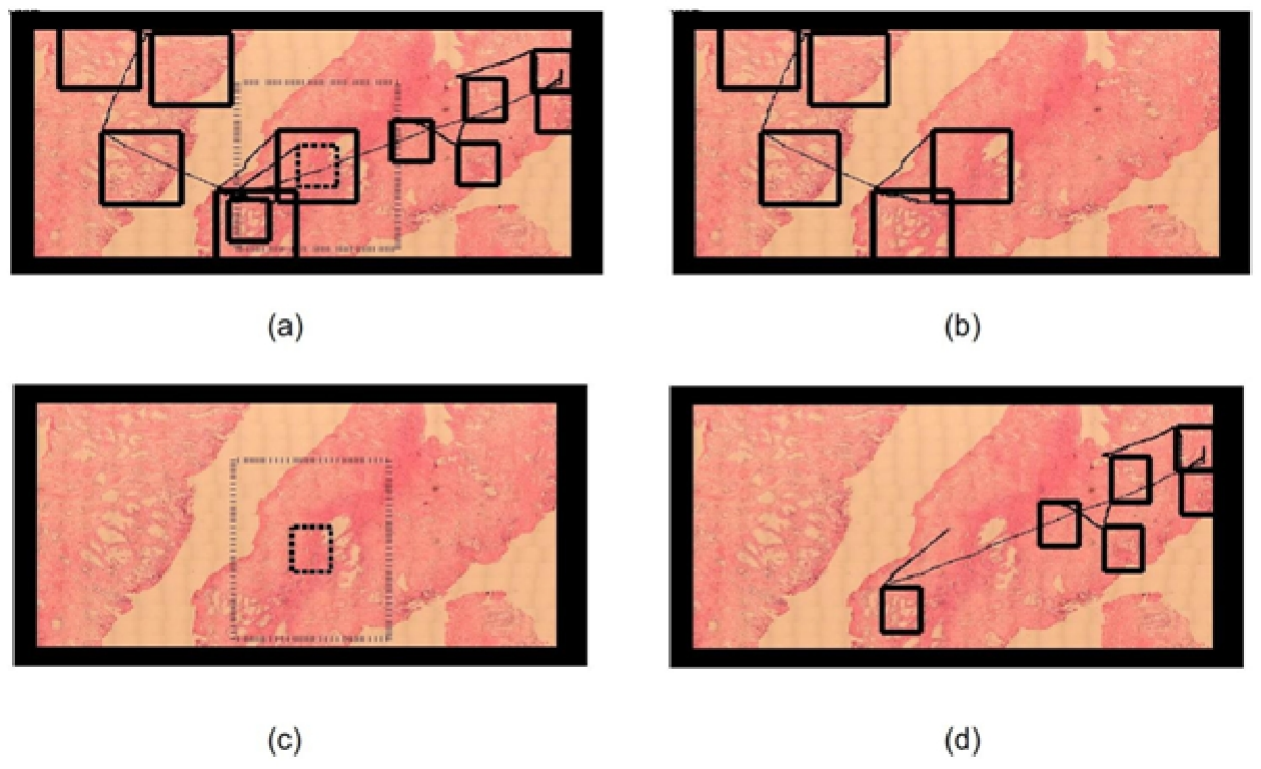
**Complete navigation example**. Complete navigation, split into four sequential panels (a, b, c and d) for the sake of understanding. Panel (a) shows the whole picture, which obviously looks very jammed by the number of windows and window movements. Hence navigation was split into a sequence shown in the next three panels: panel b starts by depicting a classical scanning pattern, composed of multiple jumps between ROIs. Panel (c) shows the magnification change from the dotted window to the thick one. Finally, the expert uses this magnification for exploring the rest of virtual slide in panel (d).

By default, all navigations start at the upper-left corner using a standard 15 × 15 *μm*^2 ^microscopical field of view, which corresponded to a 100 × 100 pixel window, within the thumbnail virtual slide. In the first part of the navigation, the window of interest is displaced through the virtual slide under a drop-drag-drop paradigm, constituted as the basic operation so that differences are mainly observed in the velocity profiles with which these navigations are carried out. In addition, pathologists could change magnification during exploration, either zooming in or out. An intermediate operation is an adjustment of the field of view when changing magnifications, i.e., a window re-sizing which allows to cover the same area when resolution changes. Overall, once this new size was set, the pathologist continued the spatial exploration using the same magnification, as observed in figure 5. So far our observations indicate what has been described in the literature, that is to say, navigation is composed of two complementary strategies: scanning and magnification.

**Figure 6:**
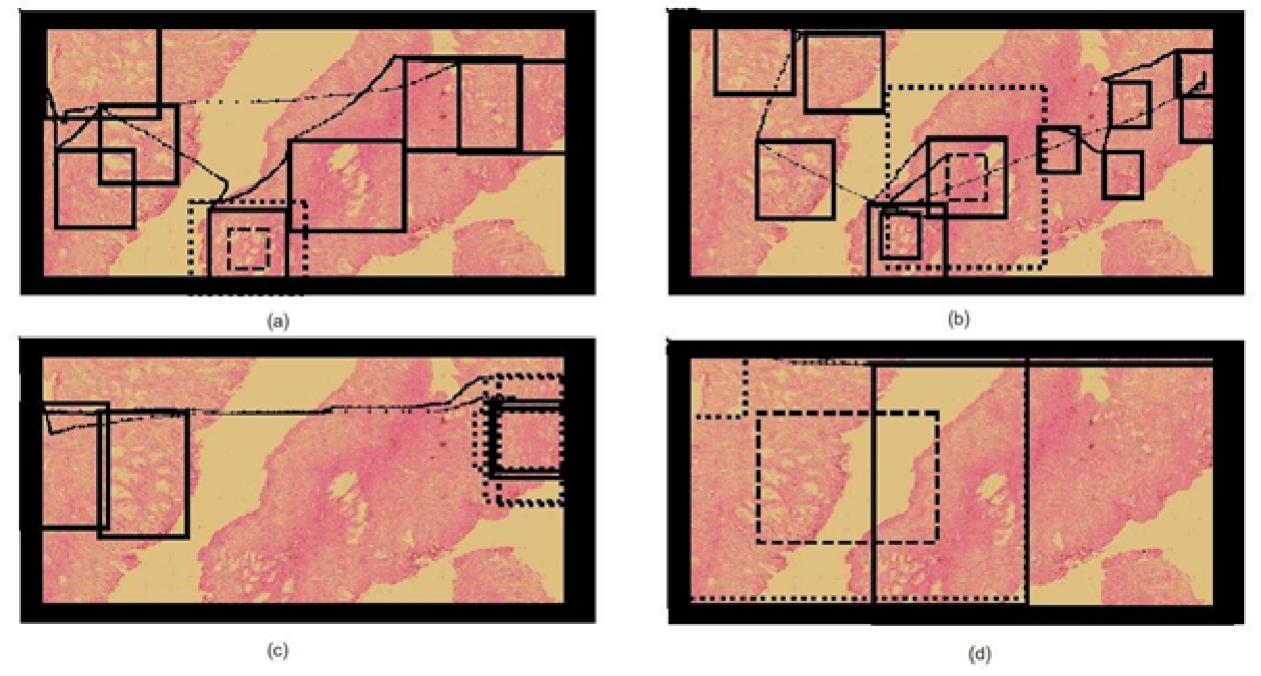
**Different navigations of each pathology over the same virtual slide**. Different navigations over the same virtual slide. In all of them the navigation pattern consists in both scanning and zooming in different ROIs. Interestingly, 3 pathologists used the default window (a, b, c) while one of them enlarged the window to cover a maximum surface.

When comparing the navigations available over the same image, a main conclusion is that every pathologist always uses both scanning and zooming operations. Interestingly, the coincidence level in the image in figure 6 was 63%, a fact that definitely suggests that the image contents steers the resultant navigation profile. The virtual slide corresponds to a prostate sample, with different sections and ROIs defined by the loci with high gland density. Figure 6 shows the four different navigation profiles, with very different diagnostic paths and observation strategies. Overall, virtual slide exploration was very variable, with different levels of interest with two main navigation patterns: a first strategy defined by three of the pathologists who used the default window and run over the virtual slide with occasional magnification changes, while in a second strategy, the pathologist enlarged the initial window to cover the maximum surface while exploring the slide. The second strategy wastes much more computational and network resources because when using larger areas, the system has to load more information and in consequence it takes more time. In these conditions, the problem is that nothing can ensure that the level of interactivity may lead to diagnosis in minimal time.

Figure 6 shows different navigations over the same virtual slide. In all of them the navigation is composed of a scanning phase and zooms, at different ROIs. Interestingly, three pathologists used the default window (a, b, c) and the fourth one enlarged the window to cover a maximum area.

**Figure 7:**
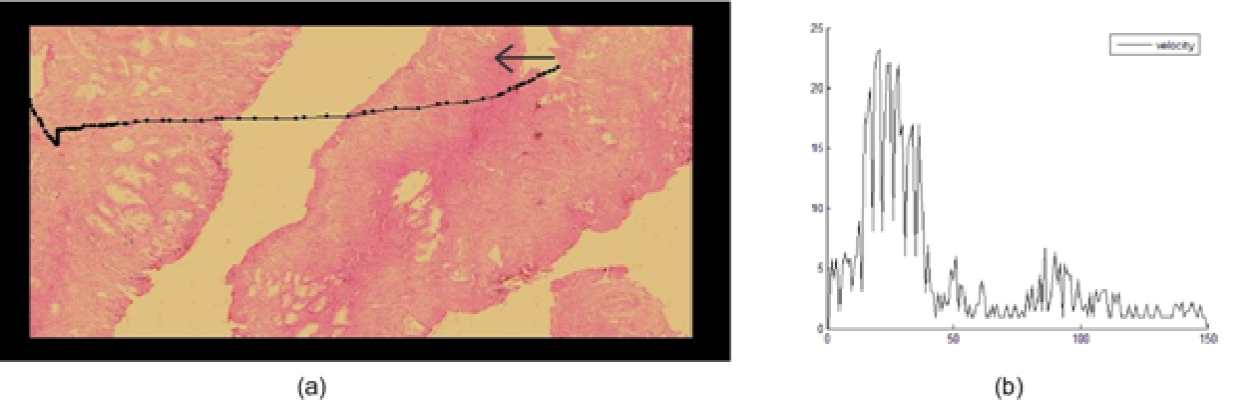
**Example of a characteristic velocity pattern**. Example of a characteristic velocity pattern generated by a left to right movement.

Finally, the velocity profiles, corresponding to the navigation displacements of the group of pathologists was observed, showing that although every pathologist has different navigation patterns, there exists a common velocity profile, i.e., velocity rapidly increases up to a certain level and then it decays with lower slopes. This profile is likely a complex mix of associated factors such as the microscopical magnification, the neuromuscular mechanics and the type of restriction demanded by the developed GUI, i.e., a drop-drag-drop sequence (screen and mouse). As shown in figure 7, explorations show high velocity profiles when experts are moving between ROIs, and lower velocity when approaching them. The GUI design allows to easily jumping from one information zone to a next, an effect observed in terms of velocity as the increasing part of the peak while a new zone is reached and a decreasing velocity profile since this zone deserves a certain amount of time for examination.

#### Path linearity among ROIs

The average coefficient of linearity is herein defined as the distance between two ROIs divided by the actual run distance. This coefficient has a maximum value of 1 when there is a linear trajectory and lower when is not linear.

**Figure 8:**
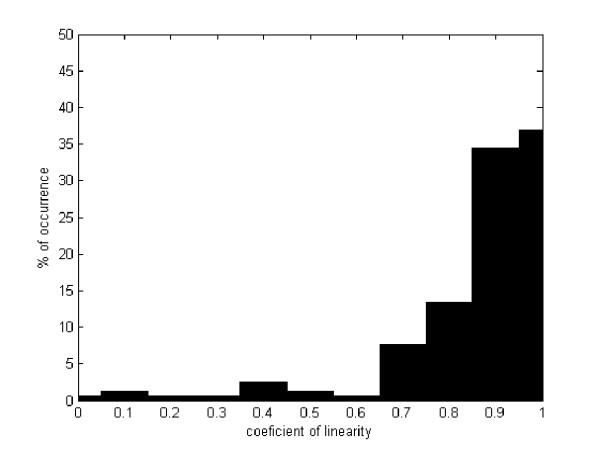
**Percentage of occurrence for each coefficient of linearity**. y-axis shows the percentage of occurrence for each coefficient of linearity in all the pathologist navigating the same image. The x-axis represents the different ranges of linearity, as observed in the figure, most of the movements are linear.

This coefficient was calculated for any trajectory between the previously determined ROIs and for every available navigation. Results are depicted in figure 8, as a histogram of occurrences in which the percentage of the available set of trajectories (157) is drawn in y-axis and the linearity coefficient in the x-axis. Interestingly, the coefficient average is about 0.8 and its standard deviation of ± 0.18, so that one can conclude that movements between ROIs are basically linear.

## Discussion

The present investigation was addressed to determine main pathologist's navigation patterns when using virtual microscopy slides, using a GUI adapted to the pathologist's workflow. Four pathologists with a similar level of experience, graduated from the same pathology program, navigated six virtual slides. Provided that a microscopical navigation is an interactive process, this study was devised to establish the relative importance of both image contents and navigation patterns of pathologists with high degree of expertise. A contribution of this work is that our GUI allowed to study not only scanning patterns, as described in the literature [2], but also magnification changes, a scenario really close to what pathologists are doing in their daily routine. Likewise, we explored the concept of ROI from different perspectives, either by analyzing the number of visits to a particular VS location as described in other previous investigations [2], and more importantly, by taking into account two new issues, i.e., the time a pathologist dedicates to explore this particular location and the coincidence level among pathologists, determinant factors which have not been evaluated so far. In addition, we also assessed the different paths described during these navigations, namely, mouse movement velocities and linearity of the diagnostic paths.

In previous studies, ROIs have been defined as areas to which the examiner is rapidly attracted to look and may contain diagnostic information [2], where visual stimulus is analyzed in detail [8] or by regions that are prioritized by the image content (first criterion) [9]. However, such definitions are still incomplete becasue a particular examiner can be rapidly drawn to regions which require a further exploration for classifying the type of information. Such pattern can be easily observed in difficult cases in which relevant information is hidden or information associated to the case is not enough as to establish an objective judgment. This leads us to acknowledge that the first path approximation is insufficient and that other criteria should be included. Classical psychophysical theory claims that the time spent in any particular task is directly related to the degree an examiner is familiar with a particular pattern [10]. We decided then to include the time spent at examining, as an evaluation criterion (second criterion) of what a ROI means in pathology, because the common pathology workflow consists in developing technical skills as to search abnormalities, a very harsh picture in many pathologies. In the present investigation, this factor resulted crucial since our GUI permitted to zoom in at any of the available preparations and so we could evaluate the importance of a particular image locus, not only because the examiner stopped there, but basically because information was valuable and required actual analysis. This fact could be established because we could compare the time spent at any of these image loci. Finally, another analysis direction (third criterion) could be the coincidence area visited during navigations, a factor which can be much more objectively included as a criterion, even though its inclusion in clinical routine is very difficult because the number of pathologists examining the same slide is very rarely larger than two. Overall, our results have supported the importance of simultaneously taking into account the three issues, mentioned before, as the base of an actual ROI definition because: (a) Pathologists are effectively attracted by some regions, as inferred from Table 1, with percentages of visited areas from 44% to 91%, a variable figure which directly depends on the diagnostic difficulty. Recall that the present study was devised for studying navigation patterns and therefore information related to the case was not available, this factor did increase the navigation time but even in these hard conditions, pathologists did not need to visit the entire virtual slide. Images associated to larger visited areas are consistent with the ones in which it was more difficult to determine the organ and/or the pathologic entity. (b) The interest for particular image loci is shared by most of the pathologists, a claim inferred from a coincidence level nearby to 70.5%, as observed in Table 2. Finally, we found that the time spent at examining each of these regions was at least a 50% of their navigation time, on specific regions previously defined, whereby some of the original regions were ruled out when considering the time factor. This evidence suggests that none of these factors could be considered as the base for defining the ROI and probably complex combinations of them are required for specific applications.

Some recent works have approached the problem of automatic ROI determination in histopathological images [11-15]. Most of these works have been devoted to segment cancer, for instance, breast tumors and colon biopsy images [13,15], since in these images the relevant regions are basically those containing abnormal architectural patterns. Nevertheless, the automatic ROI determination is still a challenging problem in many other cases in which relevant information is part of the normal patterns [14]. The problem of determining ROIs is a complex combinations of different issues, among others, the high variability at the level of the low image features (architecture, texture and color) [11], artifacts at any level of the pathology workflow chain (sampling, sections, dyes, microscopes) [16], and finally, the semantic gap between the low level image representation and the concepts formulated by a pathologists during the diagnosis process [17]. The present work proposed an alternative way to setting ROIs at taking advantage of the previously recorded navigations, i.e., information about visited area and times. There are two main applications for which determining ROIs can be fundamental: telepathology and training. In the former case, determining ROIs may accelerate the process of sending information to the specialist while in the latter, educational environments may use VS prototypes for training specialists [18]. In the case of educational applications, slides are previously navigated by a set of expert pathologists and the strategies herein proposed could be used to automatically select ROIs, without additional image processing overload. Moreover, the mouse patterns can also be used to speeding up navigations, using strategies such as cache and prefetching [7]. Finally, patterns generated during actual navigation could be used as training samples to devise automatic navigation strategies, based on machine learning [15].

Classically, exploration patterns in medical imaging have been studied by both tracking the visual system or analyzing the diagnostic path complexity [19]. However, due to the restrictions of visual systems for acquiring and interpreting high resolution images and the limited display capabilities of the computational resources [20], new alternatives for exploring virtual slides have been recently proposed [7]. In actual virtual microscopy scenarios, any diagnostic task requires the use of a device (a kind of joystick), mainly mouse devices, to point out relevant information over a low resolution version of the VS. The kind of patterns generated by the interaction of an expert and a virtual slide is obviously related to the type of interface. Overall, navigation patterns in virtual microscopy have been recently studied [21], and the few reported studies use very complicated interfaces such as eye trackers, which may bias the observed patterns. As far as we know, there exists only another study that has recorded diagnostic paths during actual navigations [21] but its analysis is totally different since therein, authors do not analyze velocities, and time is not included as a criterion at defining what a ROI is. Such study is focused on exploring prefetching and caching as possibilities to reduce navigation and transfer times. This study reported partial image covering, as observed in the present investigation, and an average linearity coefficient of 0.41 for the complete diagnostic path. Interestingly, they also found a 0.85 linearity coefficient when the analysis was carried out on three consecutive steps of the diagnostic path. On the contrary, we assessed linearity for every single step that is part of the diagnostic path, when using virtual slides in a conventional laptop and mostly under the scanning phase. Our results showed a 0.8 linearity coefficient, a quite coherent figure when comparing with the study mentioned before. In addition, we identified velocity patterns in scanning tasks, consisting in a rapid increase of velocity when pathologists leave the ROI and a decreasing profile velocity when the expert is nearby to a new ROI.

So far an optimal GUI design in virtual microscopy is still an open problem. Many virtual microscopes try to emulate the experience of navigating a real microscope, that is to say, to move a microscopical stage while zooming in and out. The simpler exploration strategy consists in using a unique window, which stands for the microscopical objective and provides interactions with a virtual stage by means of mouse panning, while the zoom operations are simulated by clicking. This strategy is of course closely related to a real microscope exploration, however if an expert might leap between two high magnification regions, that expert must zoom out from the first region, displaces the field of view to the second region and then zoom in. This pattern constitutes a natural movement with any actual microscope, but for a virtual device, it ignores the main display capacities of a virtual interface. This complex set of operations can be drastically reduced by taking advantage of both digital storage and display potentialities. The GUI herein presented approached this problem by "focus & context" [22], a set of techniques that combines a "focus view" (the auxiliary window), i.e., the GUI is partially charged of displaying the high degree of detail, and a "context view" (thumbnail window) is charged of presenting the VS at low resolution. A fundamental hypothesis in the present work for the presented design is that an expert filters information out using the "context view" and then switches to the "focus view", on which the process of information refinement and diagnostic, is achieved. Therefore, this focus view occupies most of the available area, while the smaller part for the context provides orientation during interaction. For the sake of interaction, this focus view is placed within the context view, allowing a scanning-like display as well as an additional view of the whole contents. This design compensates many disadvantages of ordinary scanning because every interaction can then be executed using only the content view so that leaps between high magnification regions become simple displacements of this focus view within the context view.

## Competing interests

The authors declare that they have no competing interests.

## Authors' contributions

LRP designed the virtual microscopy study, evaluated the results and drafted the manuscript. FG developed the algorithms and evaluated the results. ER conceived the study and participated in its design and coordination. All authors read and approved the final manuscript.
